# Prevalence of substance use disorders in an urban and a rural area in Suriname

**DOI:** 10.1186/s41182-021-00301-7

**Published:** 2021-02-02

**Authors:** Raj Jadnanansing, Matthijs Blankers, Rudi Dwarkasing, Kajal Etwaroo, Vincent Lumsden, Jack Dekker, Robbert Bipat

**Affiliations:** 1 Center for Psychiatry in Suriname and Department of Psychology Anton de Kom University of Suriname, Paramaribo, Suriname; 2 Research Department, Arkin Mental Health Institute, Amsterdam, The Netherlands; 3 Center for Psychiatry in Suriname and Faculty of Medical Science, Anton de Kom University of Suriname, Paramaribo, Suriname; 4 Research Department, Arkin Mental Health Institute Amsterdam and Department of Clinical Psychology, VU University, Amsterdam, The Netherlands; 5 Department of Physiology, Faculty of Medical Science, Anton de Kom University of Suriname, Paramaribo, Suriname

**Keywords:** Alcohol use disorder, AUDIT, Treatment gap, Suriname, Population-based study

## Abstract

**Background:**

Alcohol use disorders (AUD) have the worst impact in low-middle-income countries (LMICs), where the disease burden per liter of alcohol consumed is higher than in wealthy populations. Furthermore, the median treatment gap for AUDs in LMICs is 78.1%. The highest prevalence of AUDs worldwide in 2004 was found in the western Pacific region, Southeast Asia, and the Americas. The main aim of this study was to estimate and compare the prevalence of risky alcohol use and the extent of the treatment gap in a rural (Nickerie) and in an urban (Paramaribo) area in Suriname, a LMICs country with a wide variety of ethnic groups.

**Methods:**

The respondents were randomly recruited using a specific sampling method of the National Census Bureau. The final samples were 1837 households for Paramaribo and 1026 for Nickerie, reflecting the populations in both regions. The Alcohol Use Disorder Identification Test (AUDIT) and the Alcohol, Smoking and Substance Involvement Screening Test (ASSIST) were used to assess the likelihood of the presence of alcohol use disorder. A score of > 7 for the AUDIT implies risky alcohol use.

**Results:**

The results indicated that 2% of the women and 15% of the men in the rural area scored 8 or higher on the AUDIT. In the urban area, these numbers were 3% and 17%, respectively. In both samples, the men had the highest addiction risk at about 16% compared with 2% for females. Married persons are significantly less likely to become alcoholic than singles and other groups in Paramaribo. In both areas, higher education was associated with a lower probability of alcohol abuse and dependence, while handymen showed a higher odd. A treatment gap of 50% was found for alcohol use disorders in the rural area. The corresponding gap in the urban area was 64%.

**Conclusions:**

Surinamese men show a high prevalence of the likelihood of AUD. In addition, the treatment gap for these possible patients is large. It is therefore of paramount importance to develop therapeutic strategies with the aim of tackling this physically and mentally disabling disorder. Tailored E-health programs may be of benefit.

## Introduction

Francis Scott Fitzgerald once said: “First you take a drink, then the drink takes a drink, then the drink takes you” [1]. It is difficult to imagine a world without alcohol due to its legal and socially accepted status, as well as the pleasant effects on the consumer. People accept alcoholic beverages worldwide as an accepted part of many recreational and ceremonial activities. Although most use alcohol responsibly, some are vulnerable and become addicted to or dependent on alcohol throughout their lives. The World Health Organization (2018) describes alcohol as a psychoactive substance, with the chemical name ethanol, which can lead to addiction. In this article, we define alcohol use according to the World Health Organization’s International Classification of Diseases [2]*:* a pattern of psychoactive substance use that causes damage to health at the personal, familial, social, physical [3, 4], or mental levels [5].

Alcohol consumption and problems related to alcohol vary widely around the world but the burden of disease and death remains significant in most countries [2]. This is of particular concern in the WHO region of the American continent, which consists of at least 35 countries and more than a billion inhabitants. The consumption per capita in this region is estimated to be 30% higher than the global average. In addition, binge drinking has increased among both boys and girls over the past 5 years in this region [2].

Other substances apart from alcohol—like cocaine, cannabis, tobacco, and even sleeping pills or sedatives—seem to cause addiction worldwide as well. Drug abuse is an intense and often intentional misuse of drugs [6]. As with alcohol, the abuse of drugs can also lead to dependence or addiction. The collective term for the abuse of alcohol and drugs is substance use disorder and its epidemiology seems to be dependent on cultural values, beliefs, and attitudes to drug use, which are quite variable across cultures and geographical regions [6].

Various drugs can be abused, including illegal drugs (such as heroin and cannabis), prescription medicines (pain killers, sleeping pills), and other over-the-counter drugs (antitussive agents) that can be bought in supermarkets. The World Health Organization (2014) defines substance abuse as “the harmful or hazardous use of psychoactive substances, including alcohol and illicit drugs” [7].

Alcohol consumption represents the principal risk factor for disease and disability in middle-income countries [2]. The current article focuses on Suriname, a South American country categorized by the World Health Organization as a low-middle-income country. The 2013 Suriname National Household Drug Prevalence Survey [8] showed that alcohol was one of the most commonly used psychoactive substances in Suriname. Sex differences in alcohol use and health outcomes in middle-income and low-income countries are often found to be larger than in high-income countries [9]. The annual prevalence of alcohol use disorders is estimated at 3.4% (women 1.7%, men 5.2%) in high-income countries in Europe [10].

Suriname is a true multi-cultural society: the ancestors of the Surinamese people came from all over the world. The different population groups in Suriname live side by side peacefully in ten geographically defined areas called districts. The two areas where the current study was performed are the urban district Paramaribo and the rural district Nickerie. Rates of alcohol consumption and alcohol use disorders (AUD) can vary depending on geographic location and socio-economic circumstances. For instance, urban drug abusers are more likely to use cocaine and heroin, while rural drug abusers report more alcohol, opiate pain reliever, and stimulant use [11]. It has been concluded in the past that living in a rural area was a protective factor against alcohol dependence. However, current research data suggest a positive correlation between living in a rural area and alcohol abuse [12].

The prevalence of drug use measured with the Composite International Diagnostic Interview Version 3.0 (CIDI 3) was about 5.8% in 1916 and about 5.6% in 2010/2012 in the Netherlands [13]. The prevalence of alcohol abuse and dependence was 4.6% in both studies. Male and young participants had a higher risk of substance abuse disorder, especially those with a lower education level, those who were single, those with low incomes, and those living in the larger cities [13]. A recent study in the Netherlands with the Alcohol Use Disorder Identification Test (AUDIT) revealed that 16.8% of the population engaged in risky alcohol use; the rate was 24.1% for men and 8.7% for women. The prevalence of risky alcohol use in respondents younger than 40 was about 25%, and 11% for respondents above 40. In addition, in a high-income country like the USA, the 12-month high-risk drinking rate was estimated to be about 12.6% in 2012–2013. A recent WHO report [2] concluded that alcohol dependence and harmful use were most prevalent in high-income countries (4.5% of adults had alcohol dependence and 3.9% engaged in harmful alcohol use). In lower-middle-income countries, these percentages were 1.2% for alcohol dependence and 2.4% for harmful use. Another report states that alcohol consumption in the future will increase most sharply in the low-middle-income countries (LMICs) [14].

People with AUDs in LMICs remain untreated because they seek help for early alcohol-related problems from primary health care providers who are not trained to recognize the problem, [15] and alcohol-related problems are first addressed only when they are already severe and difficult to treat. Secondary prevention in earlier stages of drinking problems is virtually nonexistent, and many heavy drinkers who are at risk of developing an AUD in the future are not targeted by health interventions in LMICs [15]. The treatment gap for AUDs is a worldwide problem [16] and it ranges from around 50% [17] in industrial countries to 78% in LMICs [18]. The reasons vary from simple neglect to stigma and physical and sociopsychological factors [11, 15, 18].

The present study compared the prevalence of harmful alcohol use, the associated factors, and the treatment gap (respondents at risk but not in treatment) in a rural and an urban area of Suriname.

## Methods

### Study design

We conducted a large-scale investigation of substance abuse among the populations of Nickerie in 2015, and a similar investigation in Paramaribo in 2016. A large-scale study of mental health had not been performed previously in Suriname. Our study covered 1837 households in Paramaribo and 1026 in Nickerie. This number was determined in close collaboration with the *Algemeen Bureau voor de Statistiek* (ABS) of Suriname (General Bureau of Statistics), where researchers and others can obtain adequate statistics.

### Setting

The Center for Psychiatry in Suriname (PCS) in Paramaribo initiated this large-scale survey in 2014 in collaboration with the VU University Amsterdam and Arkin in the Netherlands in the context of a twinning project, with Arkin funding the study [19]. Suriname, a former Dutch colony, gained its independence and became a republic in 1975. Dutch is the official language but almost all inhabitants speak another language depending on the origin of their ancestry. The country has a population of around 600,000. In addition, almost 400,000 people from at least three generations live in diaspora in the Netherlands. The result is that there is intensive bilateral travel between the two countries.

For the purpose of the survey, two districts were selected from all the districts of the country as a whole (Paramaribo, Wanica, Nickerie, Coronie, Saramacca, Commewijne, Para, Marowijne, Brokopondo, and Sipaliwini). Paramaribo, the country’s capital, can be described as an urban, generally industrial, area, and Nickerie is a rural, mostly agricultural, area.

### Participants/respondents

The respondents were recruited in the two areas using a specific sampling method which was developed by ABS using a large sample of 10% of all households in each participating resort, which are smaller population units in each district. For large-scale research, ABS always establishes random samples for researchers. This is the most common way for researchers in Suriname to obtain a representative population for their research. The ABS selected the addresses of the respondents so as to ensure a balanced geographical distribution. The final samples consisted of 1837 households for Paramaribo and 1026 for Nickerie. This ratio reflects the relative number of households in the two areas. We expected to find 6% risky alcohol use in Paramaribo and 10% in Nickerie. To achieve a power of 0.95 (*p* = .05), a projected sample size of *n* = 2600 was calculated (two-sided tested) using an online calculator (https://www.stat.ubc.ca/~rolling/stats/size/n2.html). In addition, we expected a response rate of at least 50%. Finally, we oversampled by about 10% to account for missing data, and this was easier in Paramaribo.

In the 23rd and the 42nd week of 2016, interviewers approached approximately 1100 and 2000 participants in Nickerie and Paramaribo, respectively. The respondents were required to give both written and oral consent for the study because not all of them were literate. The respondents who ultimately agreed to participate in the study were asked to complete a confidentiality form in order to safeguard their privacy. The interview was conducted in situ in a quiet location where the interviewer explained the aim of the study and asked the questions in the respondent’s preferred language. The respondents were given enough time to complete the questionnaire and all interviews were collected and submitted electronically. Government authorities and the district police made protection arrangements for the interviewers.

### Assessment/instruments

The prevalence of alcohol consumption and abuse was assessed with two self-report questionnaires: the Alcohol Use Disorder Identification Test (AUDIT) and the Alcohol, Smoking and Substance Involvement Screening test (ASSIST).

### AUDIT

The AUDIT is a ten-item screening test for problematic alcohol use [20] developed by the World Health Organization. Items are scored on a 5-point Likert frequency scale, 0 = not at all to 4 = daily or almost daily. The total score has a theoretical range of 0 to 40. A score of 0 to 7 indicates moderate drinking, while 8 to 15 indicates an increased risk of problematic alcohol consumption. A score of 16 to 19 points indicates high risk and 20 to 40 points an extreme likelihood of addiction and abuse. These values have been validated for industrialized countries but it is the first time that this kind of research has been performed for a multi-ethnic society with specific socio-economic characteristics such as Suriname. The current study used the cut-off of 8 or more as a measure for an increased risk of problematic alcohol consumption [2].

### ASSIST

The ASSIST is a screening instrument for alcohol, smoking, and other substance use. The instrument was developed by the World Health Organization (WHO) and an international team of researchers specialized in substance use. The ASSIST consists of nine questions about drug use at any moment and the respondents were also asked specifically for each substance covered by the questionnaire whether they had ever used that substance.

### Treatment gap

After the assessment of the AUDIT, respondents were asked whether they had visited their family doctor or a hospital for the physical or mental health problems identified in the assessment. The treatment gap was calculated by estimating the percentage of subjects found to have an alcohol use disorder who did not seek help for physical or mental disorders related to alcohol use.

### Selection and training of interviewers

Before data collection began in Nickerie and Paramaribo, a small pilot study with thirty respondents was conducted in the regional health center to validate the research tool. After each day of collecting data in this pilot study, the group evaluated the difficulties they encountered, and completed questionnaire sheets with invalid or missing information were stored in a safe place.

The pilot study showed that the questionnaire had to be translated. Although Dutch is the principal language in both Nickerie and Paramaribo, the multi-cultural society of Suriname means that the inhabitants speak different languages and so the questionnaires sometimes had to be translated to make completion easier for respondents who spoke another language. Another conclusion to emerge from the pilot study was that the students who participated as interviewers required professional training. Before starting training, the students went through a selection process in which they were interviewed individually by psychiatrists.

That selection process involved looking at the background of the students and determining whether they had any prior experience with this kind of research. Students who spoke more than one language were preferred. A total of twenty students were ultimately selected to work on the study. During the training, which was given by professional psychologists and psychiatrists, all the important terms such as alcohol abuse were discussed first and then the students were told how to administer and score the questionnaire.

In the last few days of the training, different scenarios were practiced with the students to prepare them for all types of situations in the field. The training lasted 2 weeks.

### Statistics

Differences in the demographic characteristics of Paramaribo and Nickerie, as well as the baseline characteristics of respondents with or without a risk of addiction (as stated above, AUDIT > 7) were compared using chi-squared (*χ*^2^) testing in the case of categorical variables. We used simple logistic regression to calculate the odds ratio for the demographic variables. In addition, we applied multivariable logistic regression analysis to evaluate demographic factors that may predict the risk of addiction. All statistical analyses were conducted using SPSS (version 26; IBM; NY).

SPSS and GraphPad for Prism version 8.3 were used for the quality assurance of the analyses. Data are presented as mean values (95% CI) unless noted otherwise. A *p* value < 0.05 was considered significant.

## Results

A total of 1837 participants were included in Paramaribo, 1065 women and 772 men. In Nickerie, there were 593 female participants and 433 men, bringing the number to a total of 1026. The entire study population therefore consisted of 2863 participants in the two regions.

Paramaribo has a population of 140,679 people, 51% female and 49% male, of whom 53% are younger than 40 and 47% older than 40. Nickerie has a population of 71,867 people, 47% female and 53% male, of whom 52% are younger than 40 and 48% older than 40. There was a significantly higher number of elderly women and a significant underrepresentation of younger men in both samples. After analyzing the data, we found a ratio of 58% women (*χ*^2^ = 35.6; df = 1; *p* = 0.000) in Paramaribo and 59% women in Nickerie (*χ*^2^ = 46.9; df = 1; *p* = 0.000), of whom 54% were older than 40 years (*χ*^2^ = 13.3; df = 1; *p* = 0.000) (Table 1).

**Table 1:** Demographics of the Paramaribo and Nickerie samples

Variable		Total	Paramaribo	Nickerie	***p***
Age (median (SD))		39.97 (14.2)	39.77 (14.3)	40.32 (13.8)	0.32
Gender	Female	1658 (57%)	1065 (58%)	593 (57.8%)	0.93
Education	Low	1590 (62%)	936 (60.8%)	654 (63.8%)	0.00*
	Secondary	824 (32.1%)	466 (30.3%)	358 (34.9%)	
	High	150 (5.9%)	137 (8.9%)	13 (1.3%)	
Marital Status	Single	1151 (40.2%)	877 (47.7%)	274 (26.7%)	0.00*
	Married	978 (34.2%)	484 (26.3%)	494 (48.2%)	
	Widow	104 (3.6%)	62 (3.4%)	42 (4.1%)	
	Divorced	133 (4.6%)	75 (4.1%)	58 (5.7%)	
	Concubinate	443 (15.5%)	296 (16.1%)	147 (14.3%)	
	Long-distance relationship	53 (1.9%)	43 (2.3%)	10 (1.0%)	
Number of children		1.36 (1.9)	1.47 (2.1)	1.16 (1.8)	0.00*
Ethnic background	West Indians	1058 (37%)	424 (23.1%)	634 (61.9%)	0.00*
	Creole	626 (21.9%)	531 (28%)	95 (9.3%)	
	Maroon	222 (7.8%)	216 (11.8%)	6 (0.6%)	
	Javanese	368 (12.9%)	187 (10.2%)	181 (17.7%)	
	Mixed	474 (16.6%)	395 (21.5%)	79 (7.7%)	
	Different	113 (3.9%)	84 (4.6%)	29 (2.8%)	
Daily activity	Student	328 (11.5%)	234 (12.7%)	94 (9.3%)	0.00*
	Working part-time	172 (6%)	104 (5.7%)	68 (6.7%)	
	Working full-time	1274 (44.7%)	939 (51.1%)	335 (33%)	
	Unemployed/jobseeker	212 (7.4%)	111 (6%)	101 (10%)	
	Homemakers	602 (21.1%)	274 (14.9%)	328 (32.3%)	
	Handyman	83 (2.9%)	38 (2.1%)	45 (4.4%)	
	Retired	181 (6.3%)	137 (7.5%)	44 (4.3%)	

*p < 0.05 for ratio. Low education is up to primary school, secondary education is up to high school, and high education is comparable with university or college degree

We can therefore conclude that the samples were not significantly different in terms of age and gender than the populations of the two areas. However, we saw significant differences in marital status, number of children, ethnic background, and daily activity. In Nickerie, we found more married couples and fewer single people, more respondents with few children, and more with an Indian or Indonesian background. Furthermore, we found fewer respondents with a full-time job and more homemakers.

The first questions in the epidemiological study were the nine questions from the ASSIST questionnaire. The respondents were asked specifically whether they had used the substances covered by the questionnaire. Table 2 shows the use percentages for each sample and the test quantities according to the possible differences between the samples.

**Table 2:** Differences between Paramaribo and Nickerie regarding substance use

Question	Paramaribo	Nickerie	***p***
	Yes	Yes	
1. Have you ever used tobacco products (cigarettes, cigars, tobacco, etc.) in your life?	820 (44.6%)	376 (36.6%)	0.000*
2. Have you ever used alcoholic beverages (beer, wine, spirits, shooters, alcopops, mixed, drinks etc.) in your life?	1396 (76%)	738 (71.9%)	0.017*
3. Have you ever used cannabis (marijuana, weed, hash, etc.) in your life?	247 (13.4%)	73 (7.1%)	0.017*
4. Have you ever used stimulants (type amphetamine) in your life?	29 (1.6%)	7 (0.7%)	0.039*
5. Have you ever used cocaine (coke, crack, base coke, etc.) in your life?	33 (1.8%)	20 (2%)	0.768
6. Have you ever used volatile snuff/inhalation in your life?	36 (2%)	7 (0.7%)	0.070
7. Have you ever used sleeping aids and tranquilizers in your life?	230 (12.5%)	102 (10%)	0.040*
8. Have you ever used hallucinogens (LSD, mushrooms, PCP, ketamine, special K, mescaline) in your life?	11 (0.6%)	1 (0.1%)	0.046*
9. Have you ever used opiates (heroin, morphine, methadone, subutex, suboxone, buprenorphine, codeine) in your life?	11 (0.6%)	38 (3.7%)	0.000*

*p < 0.05 for ratio

Table 2 indicates that respondents from Paramaribo stated significantly more often that they had used some kind of substance in the past—with the exception of cocaine, opiates, and volatile stuff—than those in Nickerie. In both samples, approximately 74% of the respondents had used alcohol; 44.6% in Paramaribo and 36.6% in Nickerie had used tobacco. These were therefore the most widely used substances.

The addiction risk determined by the Alcohol Use Disorders Identification Test (AUDIT) was 6.4% in Paramaribo and 5.8% in Nickerie. This is not a significant difference. In both areas, most respondents (about 93%) were not at risk (AUDIT score 0–7). A group of about 6% had a medium risk (AUDIT score 8–15) and about 1% a high risk or probably addiction and abuse (AUDIT score > 15).

Tables 3 and 4 give an overview of the risk of addiction in the two samples for the different demographic categories. In Paramaribo, the risks for younger respondents (11%) and the male respondents (16.5%) were significantly higher with regard to age and gender. The respondents with the highest education had the lowest risk (2.9%) among all education levels. Single and divorced people had the highest risks (10.4% and 9.3%) for civil status and respondents with a Javanese background had the lowest risk (3.2%) when comparing ethnic background. The unemployed respondents and the handymen (a person employed to do occasional domestic repairs and minor renovations) had the highest risks (13.5% and 26.3%) among the occupations. In Nickerie, we saw three significant differences: the male respondents had the highest risk (15%), as did respondents with a mixed background (15.5%). People with a Javanese background had the lowest risk (3.2%) and the respondents working full-time and the handymen had the highest risks (10.1% and 28.9%). In subsequent analyses, we calculated that, in the age group under 40 years, the risk of addiction was about 18.5% in men and about 3% in women (in Paramaribo and in Nickerie). In the age group above 40 years, the risk of addiction was about 12.5% in men and about 1.3% in women. The risk for addiction is therefore particularly high in younger men, but not in women.

**Table 3:** Overview of the risk for addiction in the two samples for the different demographic categories

Variable		Paramaribo	***p***	Nickerie	***p***
		Risky alcohol use		Risky alcohol use	
**Age**	< 40	101 (11%)	0.000*	39 (8.3%)	0.334
	> 40	53 (5.8%)		37 (6.7%)	
**Gender**	Male	127 (16.5%)	0.000*	65 (15%)	0.000*
	Female	27 (2.5%)		11 (1.9%)	
**Education**	Low	91 (9.7%)	0.000*	54 (8.3%)	0.164
	Secondary	41 (8.8%)		20 (5.6%)	
	High	4 (2.9%)		2 (15.4%)	
**Marital status**	Single	91 (10.4%)	0.000*	23 (8.4%)	0.072
	Married	24 (5%)		32 (6.5%)	
	Widow	1 (1.6%)		0 (0%)	
	Divorced	7 (9.3%)		8 (13.8%)	
	Concubinate	21 (7.1%)		11 (7.5%)	
	Long-distance relationship	10 (23.3%)		2 (20%)	
**Ethnic background**	West Indian	36 (8.5%)	0.027*	50 (7.9%)	0.021*
	Creole	46 (8.7%)		9 (9.5%)	
	Maroon	21 (9.7%)		0 (0%)	
	Javanese	6 (3.2%)		6 (3.3%)	
	Mixed	32 (8.1%)		5 (6.3%)	
	Different	13 (15.5%)		6 (20.7%)	
**Daily activity**	Student	25 (10.7%)	0.000*	3 (3.2%)	0.000*
	Working part-time	13 (12.5%)		9 (13.2%)	
	Working full-time	81 (8.6%)		34 (10.1%)	
	Unemployed/jobseeker	15 (13.5%)		6 (5.9%)	
	Homemakers	3 (1.1%)		7 (2.1%)	
	Handyman	10 (26.3%)		13 (28.9%)	
	Retired	7 (5.1%)		4 (9.1%)	

*p < 0.05

**Table 4:** Multivariable logistic regression for demographic characteristics associated with alcohol use disorder for both regions

			OR	Lower 95% CI	Upper 95% CI	***p*** value
**Step 1**^**a**^	**Gender**	Female	0.149	0.096	0.229	0.000
	**Age**	< 40	1.741	1.210	2.506	0.003
	**Education**	High (ref)				
		Low	3.146	1.318	7.506	0.010
		Secondary	2.673	1.097	6.514	0.030
	**Marital status**	Long-distance relationship (ref)				
		Single	0.353	0.166	0.753	0.007
		Married	0.297	0.130	0.680	0.004
		Widow	0.097	0.011	0.846	0.035
		Divorced	0.550	0.207	1.459	0.229
		Concubinate	0.274	0.119	0.635	0.003
	**Ethnic background**	Different (ref)				
		West Indian	0.474	0.252	0.892	0.021
		Creole	0.478	0.247	0.922	0.028
		Maroon	0.598	0.278	1.286	0.188
		Javanese	0.173	0.075	0.398	0.000
		Mixed	0.443	0.221	0.888	0.022
	**Daily activity**	Retired (ref)				
		Student	0.720	0.301	1.721	0.460
		Working part-time	1.005	0.420	2.407	0.991
		Working full-time	0.913	0.440	1.897	0.808
		Unemployed	0.969	0.416	2.256	0.941
		Homemakers	0.463	0.169	1.267	0.134
		Handyman	2.932	1.237	6.946	0.015
Intercept			0.343			0.149

^a^Variables entered in step 1: gender, age, education, marital status, ethnic background, and daily activity

We used univariate logistic regression to calculate the odds ratios for addiction risk on the basis of education level, civil status, ethnic background, and daily activities for both men and women in both areas. We saw no significant differences in women for any of the categories in Paramaribo or Nickerie. We did find a significantly lower odds ratio for men with an Indonesian background (OR, 0.143; 95% CI, 0.036–0.574; *p* = 0.006) in Nickerie than in the other groups. Male respondents with a lower or secondary education level had a higher OR (OR for low education, 4.971; 95% CI, 1.52–16.23; *p* = 0.008; OR for secondary education, 5.54; 95% CI, 1.64–18.71; *p* = 0.006) than those with a higher education level in Paramaribo. There was no difference between single men and those in a long-distance relationship. Men who were married, divorced, or living with a partner all had a lower OR than those in a LAT relationship (OR married, 0.214; 95% CI, 0.084–0.0541; *p* = 0.001; OR divorced, 0.269; 95% CI, 0.078–0.9362; *p* = 0.039; OR living together, 0.323; 95% CI, 0.124–0.843; *p* = 0.021).

All the demographic variables are significant predictors of addiction risk. However, predictive power is low (Cox and Snell *R* square 0.096 and Nagel *R* square 0.22).

Table 5 shows that 34 of a total of 154 respondents with a risk of addiction in Paramaribo, and 25 of 77 respondents with a risk of addiction in Nickerie, were receiving treatment for mental disorders. The treatment gaps were about 82% for Paramaribo and about 74% for Nickerie, with no significant difference (*χ*^2^ = 2.4; df = 1; *p* = 0.12). We did not assess those who sought treatment for physical but not mental disorders due to alcoholism, or vice-versa.

**Table 5:** Treatment (mental and physical) seeking for alcohol abuse and dependence

	Nickerie	Paramaribo
Total AUDIT > 7 and < 20	77	154
Number of treatment seeking (%)	22 (31%)	29 (20%)
Total AUDIT > 19	6	11
Numbers seeking treatment (%)	3 (50%)	5 (45%)

## Discussion

The two samples in this epidemiological study were representative for the general population in terms of sex and age. However, in the urban area, there were fewer single and more married respondents, more Creole and Maroon respondents, fewer West Indian and Javanese respondents, more respondents who work full-time, and fewer housewives. These significant differences are not entirely in line with the population structure in terms of sex and age of the two areas: there was a slight but significant overrepresentation in both samples of older women and a slight but significant underrepresentation of younger men.

Alcohol use disorder was about the same in both areas, 6.4% in the urban area and 5.8% in the rural area. So our expectation that the addiction risk in the rural area would be higher was not confirmed [12]. Approximately the same percentages are found in the Netherlands. However, in the latest WHO report on alcohol in the world, researchers concluded that the prevalence rates in lower-middle-income countries were 1.2% for alcohol dependence and 2.4% for harmful use [2].

An addiction risk of about 6% in Suriname is therefore comparable with the Netherlands but not with lower-middle-income countries [21]. An increased emphasis on this high risk from the Surinamese mental health care system would therefore seem to be appropriate.

In both samples, the addiction risk was higher for men (at about 16%) than for females (2%). The odd ratios for the females in both areas were about the same for all the demographic features. We found that, in the case of men in the rural area, the lowest risk was in Javanese males. In the urban area, the risk factors were low/secondary education, single, and widower. These findings also concur with foreign results. The percentages of risky alcohol use found were lower in the areas we studied than in the population of the Netherlands. Risky alcohol use was found in approximately 17% of Surinamese males compared with 24.1% of Dutch males. Risky alcohol use was seen in approximately 2% of Surinamese women as compared with 8.7% of Dutch women [22].

Predictive power for addiction risk was low. The higher prevalence of risky alcohol use in men is in complete accordance with international trends [2]. This is also true for the high prevalence in younger males and a lower but substantial prevalence above age 40 in male subjects [2], a pattern that has mainly been observed in the USA [23–25].

According to the World Health Organization, the male/female ratio for heavy drinking in low-middle-income countries is about 2.6 [2]. Our study found a higher odds ratio. A possible explanation could be that drinking in women is socially less acceptable in these societies, which consist mainly of descendants of people from Asia and Africa who have generally tried to maintain their cultural habits [20, 26–28]. However, the low prevalence at older ages in women contrasts with the results found in a study conducted in the Netherlands, where this rate increases with age and is highest in the older age groups of 40 years and more [21].

This study clearly showed the effect of marital status and education level. Married persons are significantly less likely to become alcoholic than singles and other groups in Paramaribo. The effect of marriage in a study with twins has shown that sustained intimate relationships are associated with less alcohol consumption [29]. The reason we did not find clear evidence of this phenomenon in Nickerie remains obscure. In both areas, a lower risk of alcohol abuse and dependence was found to be correlated with higher educational level. A household study in the USA showed that a higher educational level was linked to a lower probability of alcoholism [30]. The same study also concluded that ethnicity could mitigate the education effect and this could explain the slight deviations in the findings for Nickerie.

The finding of the higher risk for handymen is also found in other studies and it can be associated with educational level [31]. Furthermore, an American study found an OR of 1.37, CI  =  1.09 to 1.73 for Amerindians [32]. This is certainly a major public health concern for this group of people [33] and, given our results indicating high prevalence among the comparable indigenous group in Suriname, it merits more attention.

Another remarkable result of this study is the treatment gap of about 80%. This was also observed in other low-middle-income countries like Ethopia, Nepal, and India [2, 34]; the significant gap in the two areas we studied makes this an area about which our society should be concerned.

## Conclusions

As the results indicate, alcohol use in Nickerie and Paramaribo is problematic in young male individuals (but not females) and there is a treatment gap. To remedy that gap, it is important for those suffering from problematic alcohol use to know where to turn for help. First of all, doctors and nurses in primary health care should be made aware of this gap and trained appropriately to deal with the problem. Secondly, e-health programs have been shown to be very effective in remote areas like Nickerie [35, 36]. These programs use mobile and internet-based devices to offer assistance to people needing treatment and facing either physical or social barriers.

An appropriate assessment will have to be made to determine whether these approaches are useful for younger men (especially those without work, with a lower education and/or working as handymen) in the Surinamese situation. Research in this area is warranted.

## Data Availability

The data that support the findings of this study are available from the Center of Psychiatry in Suriname (PCS) but restrictions apply to the availability of these data, which were used under license for the current study and so are not publicly available. Data are, however, available from the authors upon reasonable request and subject to permission from the Center of Psychiatry in Suriname.

## Abbreviations

ABS: *Algemeen Bureau voor de Statistiek* (General Bureau of Statistics)
ASSIST: The Alcohol, Smoking and Substance Involvement Screening Test
AUD: Alcohol use disorders
AUDIT: The Alcohol Use Disorder Identification Test
CIDI 3: Composite International Diagnostic Interview Version 3.0
LMICs: Low-middle-income countries
OR: Odds ratio
PCS: The Center for Psychiatry in Suriname
WHO: World Health Organization
